# Effect of transient expression of fluorescent protein probes in synovial and myoblast cell lines

**DOI:** 10.1186/2193-1801-1-36

**Published:** 2012-10-22

**Authors:** Seiji Shibasaki, Aika Fujita, Chihiro Usui, Sachiko Watanabe, Sachie Kitano, Hajime Sano, Tsuyoshi Iwasaki

**Affiliations:** 1 Department of Pharmacy, School of Pharmacy, Hyogo University of Health Sciences, 1-3-6 Minatojima, Chuo-ku, Kobe, 650-8530 Japan; 2 Department of Internal Medicine, Division of Rheumatology, Hyogo College of Medicine, 1-1 Mukogawa-cho, Nishinomiya, 663-8501 Japan

**Keywords:** C2C12 cell, Cell viability, Fluorescent protein, MH7A cell, Transfection

## Abstract

Here, we investigate the appropriate fluorescent proteins for use in the culture of synovial MH7A and myoblast C2C12 cells. Fluorescent signal intensities of 3 different fluorescent proteins were examined in these cell lines. The fluorescent intensity of transiently expressed AcGFP, DsRed, and mStrawberry were examined in these cell lines, and the influence of the amount of plasmid used on transfection efficiency and cell viability were investigated.

## Background

Several types of tags have been used for analyses of the expression of a gene of interest or localization of proteins under investigation. Since the 1990s, green fluorescent protein (GFP) has been used to analyze the expression of genes of interest or the localization of such proteins. Jellyfish *Aequorea victoria* GFP and its variants are the most popular and representative fluorescent proteins for use as molecular probes in molecular and cell biology (Cubitt et al. 1995). Furthermore, these proteins are used to monitor bio-production in living cells and for bio-sensing of environmental conditions (Shibasaki et al. 2001). Recently, several fluorescent proteins other than GFP have also been used in biological studies (Brandariz-Nuñez et al. 2011, Piatkevich and Verkhusha, 2011).

In mammalian cell lines, there are 2 typical ways for introducing a gene of interest into host cells. One involves the integration of the gene of interest into the genome for stable expression, and the other is its introduction into host cells as a plasmid for transient expression. Usually, a single copy of the gene of interest is maintained in the host cells when the gene in integrated into the genome. In contrast, plasmids used for transient expression are typically present in multiple copies. Production of the foreign protein from a multi-copy plasmid is thought to affect metabolic load in the host cells (Khoo et al. 2007), which could result in different effects in different cell lines. Therefore, the impact of using different fluorescent proteins as molecular probes should also be examined in various kinds of host cells.

In this study, we chose 2 types of mammalian cells, MH7A synovial cells and C2C12 mouse myoblast cells, to evaluate differences in fluorescent protein production. These cell lines have frequently been used as model systems for the investigation of physiological or molecular biological studies. The MH7A cell line has been used to investigate cell proliferation, inflammation, or signaling pathways in synovial cells (Kitano et al. 2006). It has also been used to evaluate the effect of novel immunosuppressants for rheumatoid arthritis. The C2C12 cell line is a useful model for studying the differentiation of non-muscle cells into skeletal muscle cells. Treatment of these cells with bone morphogenic protein 2 causes a shift in the differentiation pathway from myoblast cells to osteoblast cells (Katagiri et al. 1994).

We chose 3 fluorescent proteins of different colors, viz., AcGFP, DsRed-monomer, and mStrawberry, for investigation in these cell lines. AcGFP is a green fluorescent protein derived from *Aequorea coerulescens* and has silent mutations that create an open reading frame almost entirely composed of preferred human codons (Haas et al. 1996). The DsRed-monomer is a monomeric mutant derived from the tetrameric *Discosoma* spp. red fluorescent protein (Matz et al. 1999). mStrawberry is a mutant protein derived from DsRed (Shaner et al. 2004).

We constructed 3 constructs to express these fluorescent proteins, using pcDNA3.1(+) as plasmid backbone. Fluorescence was observed by fluorescence microscopy and quantified using a conventional micro-plate reader. Fluorescence intensity of each of the proteins changed during cultivation of the transfected cells in a manner that differed depending on protein/host cell combinations. In addition, the effect of the amount of plasmid DNA and the effect of each fluorescent protein on cell viability was also examined.

## Results and discussion

### Plasmids and transfection of cell lines

We constructed 3 plasmids expressing fluorescent proteins of different colors under the control of the CMV promoter, i.e., pcDNA-AGF (AcGFP), pcDNA-DRD (DsRed), and pcDNA-STB (mStrawberry). After verifying the DNA sequence and purity of plasmids, they were introduced into synovial MH7A or myoblast C2C12 cells by lipofection reagent. To investigate whether 0.8 μg, the amount of plasmid DNA typically used to transfect cells in 500 μl of medium in a single well of a 24-well plate (Colapinto et al. 2006, Emonet et al. 2009), we decreased the amount of plasmid DNA for all transfections from 0.8 μg to 0.1 μg in the same volume of medium.

### Observations of fluorescent protein-expressing cells

**Figure 1:**
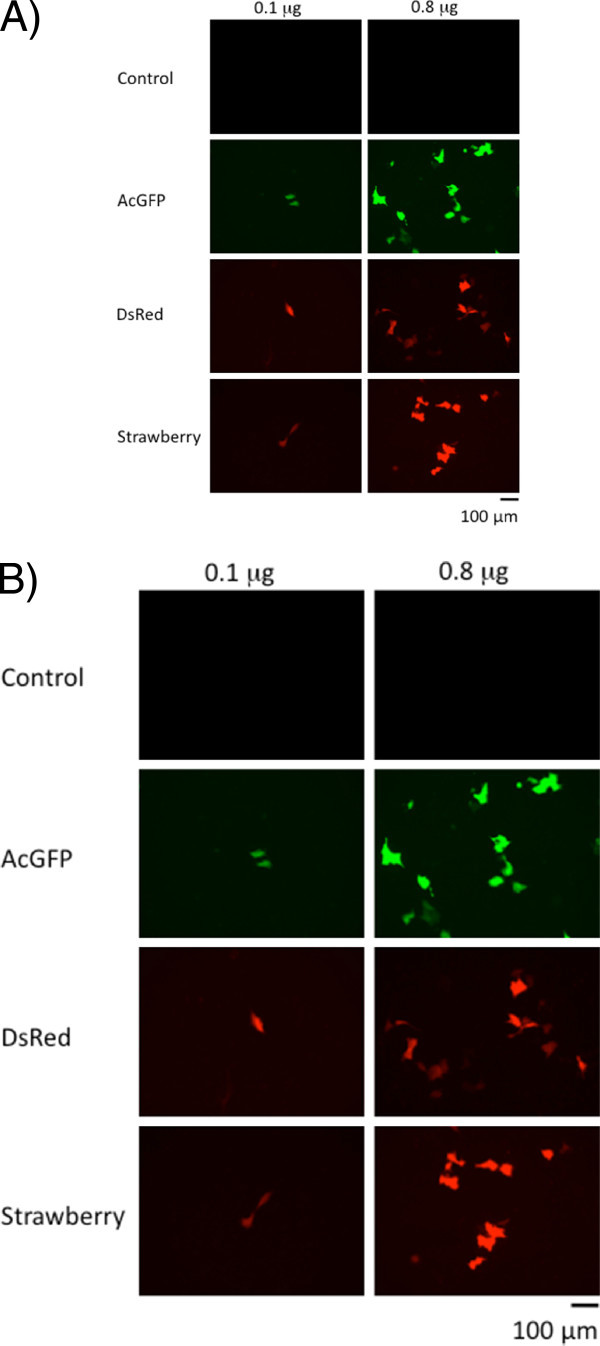
**Fluorescence micrographs of fluorescent protein**-**transfected cell lines.** Images of (**A**) MH7A cells harboring the control plasmid pcDNA3.1(+), pcDNA-AGF (AcGFP), pcDNA-DRD (DsRed), and pcDNA-STB (mStrawberry) were taken under fluorescence microscopy with a suitable band path filter. Left and right photographs represent cells transfected with 0.1 μg or 0.8 μg of plasmids, respectively. Images of (**B**) C2C12 cells were similarly taken. Bar = 100 μm.

First, MH7A cells transfected with any of the 3 above plasmids were observed using fluorescent microscopy. All 3 fluorescent proteins were clearly expressed by 2 days after transfection (Figure 1A). In wells where we had introduced 0.1 μg of DNA into MH7A cells, approximately 10% of the cells well fluoresced, compared to about 50% of cells where we had utilized 0.8 μg of DNA in the transfection. When we observed C2C12 cells transfected with these plasmids (Figure 1B), we noted that AcGFP or mStrawberry fluoresced brightly in C2C12 cells that had been transfected with 0.8 μg plasmid DNAs.

In both MH7A and C2C12 transfected cells, AcGFP and mStrawberry was clearly observed under fluorescence microscopy. Codon-optimization of these sequences for mammalian host cells (Haas et al. 1996) possibly improved the expression of these proteins in both MH7A cell and C2C12 cells. We also examined the effect of using 1.6 μg of DNAs in transfection, compared to using 0.8 μg; however, this did not lead to a marked increase in fluorescence (data not shown).

### Fluorescence intensity

We also attempted to evaluate protein expression more quantitatively, rather than merely comparing observation of fluorescence by microscopy. The fluorescence intensity of the transfected cells was measured using a multi-well microplate reader with appropriate wavelength settings at room temperature.

**Figure 2:**
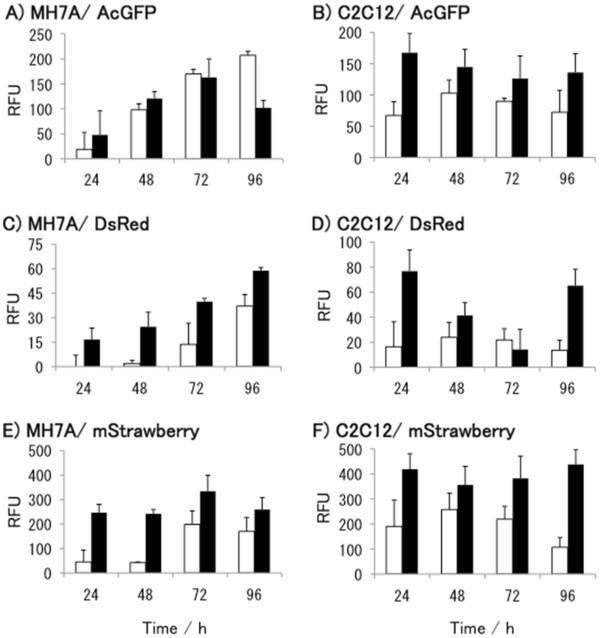
**Fluorescence intensity of the various fluorescent proteins.** Relative fluorescence units (RFU) were measured for cells transfected with AcGFP (**A**) and (**B**), DsRed (**C**) and (**D**), mStrawberry (**E**) and (**F**). Panels (**A**), (**C**), and (**E**) show the RFUs in MH7A cells, and (**B**), (**D**) and (**F**) represent those in C2C12 cells. Open bars and black bars represent 0.1 and 0.8 μg plasmid-transfected cells, respectively. Each result is expressed as the mean ± SD of triplicate experiments. The fluorescence of the control cells harboring pcDNA3.1(+) was subtracted from mean values.

The fluorescence intensities of MH7A cells and C2C12 cells expressing AcGFP (Figure 2A and B) and mStrawberry (Figure 2E and F), which showed bright fluorescence under microscopy, were much higher than that of the DsRed-monomer (Figure 2C and D). For instance, at 48 h (the time at which images shown in Figure 2 were taken), the fluorescence of AcGFP and mStrawberry in MH7A cells transfected with 0.8 μg of either plasmid (Figure 2A and E) was 5.0-fold and 9.9-fold higher than that of the DsRed-monomer (Figure 2C). Similarly, in C2C12 cells, fluorescence of AcGFP and mStrawberry (Figure 2B and F) were 5.0-fold and 8.7-fold higher than that of the DsRed-monomer (Figure 2F). The reason why the DsRed-monomer fluoresces less intensely compared with the other fluorescent proteins is not related to the molecular size of the protein, but rather to its amino acid sequence. The molecular weight of these proteins, calculated from their amino acid sequences (http://www.expasy.org/), are very similar; viz., AcGFP: 26.9 kDa, DsRed-monomer: 25.4 kDa, and mStrawberry: 26.4 kDa. Therefore, the amino acid substitutions made to DsRed not only conferred a fluorescent “color-shift” (Shaner et al. 2004) but also enhanced the stability of the resulting mStrawberry protein.

As for the amount of plasmid used in transfection of both cell lines, a greater amount results in higher fluorescence intensity, except for expression of AcGFP in MH7A cells (Figure 2A). In MH7A cells expressing AcGFP, higher fluorescence intensities were observed on transfected cells with 0.1 μg plasmid than cells with 0.8 μg plasmid at 72 h and 96 h. On the other hand, at 24 h after transfection of C2C12 cells, cells transfected with 0.8 μg plasmid of AcGFP or mStrawberry have a 2.5-fold and 2.2-fold higher fluorescence intensity, respectively, than that of C2C12 cells transfected with 0.1 μg plasmid (Figure 2B and 21F). Thus, the fluorescence intensity is not in proportion to the amount of DNA used in transfection, precisely.

### Cell viability

**Figure 3:**
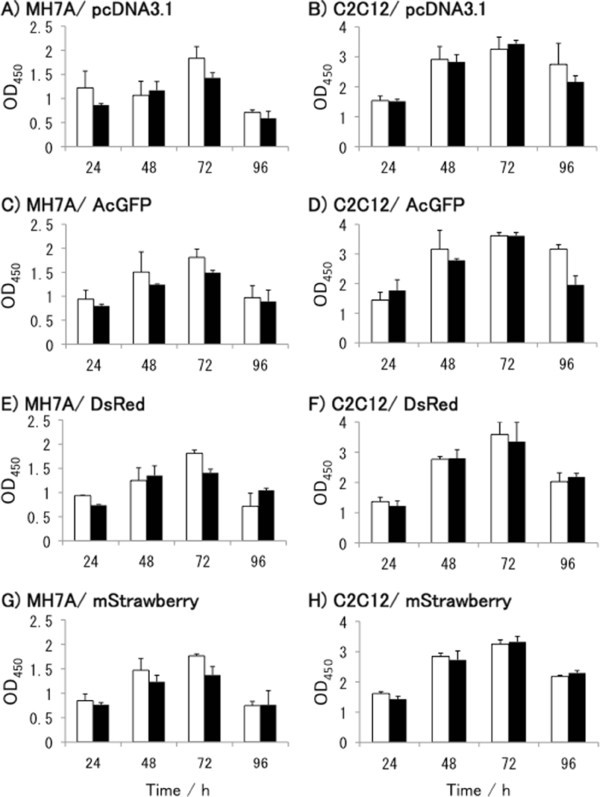
**Cell viability of transfected cell lines.** Cell viability was measured using the biochemical reaction of WST-8 within living cells. Absorbance at 450 nm (OD_450_) represents the relative viability of cells. (**A**) and (**B**) are control cells harboring pcDNA3.1(+). Cells harboring the AcGFP (**C** and **D**), DsRed (**E** and **F**), mStrawberry (G and H) plasmids are shown. Panels (**A**), (**C**), (**E**), and (**G**) represent the viability of MH7A cells, and (**B**), (**D**), (**F**), and (**H**) represent the viability of C2C12 cells. Open bars and black bars represent 0.1 and 0.8 μg plasmid-transfected cells, respectively. Each result is expressed as the mean ± SD of triplicate experiments.

Both transfected MH7A and C2C12 cell lines showed similar growth patterns (Figure 3), i.e., the maximum cell numbers were observed at 72 h during a cultivation period of 96 h. Expression of exogenous proteins from the plasmids seemed to have little effect on the cells, as there was no noticeable difference between cells harboring the control plasmid (Figure 3A and B) and those harboring the fluorescent protein-encoding plasmids in either the MH7A cell line (Figure 3C, E, and G) or the C2C12 cell line (Figure 3D, F, and H). No growth inhibition was observed when plasmid-transfected cells were compared with cells harboring no plasmids (data not shown). This is consistent with a previous report on transfection using the same plasmid backbone (Nakagawa et al. 1999). These results indicate that the plasmids and transfection reagent by themselves do not have deleterious effects in cell culture. As for the effect of the amount of plasmid on cell survival, the viability of MH7A cells transfected with 0.1 μg of DNA was 22% higher than that of that of cells transfected with 0.8 μg of DNA, at 72 h (Figure 3A, C, E, and G). Thus, using lesser amounts of plasmid may contribute to the viability of MH7A cells.

One obvious difference between the 2 cell lines was their growth rate. C2C12 cells grew around 1.5–2 times faster than MH7A cells. No toxicity of transfection was confirmed for either cell line; however, there may be differences in sensitivity to the transfection reagent, as seen when Figure 3C, E, and G are compared to Figure 3D, F, and H. Such a comparison indicates that the MH7A cell line may be more sensitive to lipofection reagent than the C2C12 cell line.

## Conclusions

We have shown that AcGFP and mStrawberry are able to function as practical fluorescent probes in MH7A and C2C12 cell lines. mStrawberry is mostly used as a fluorescence marker in a transient expression system, using pcDNA3.1(+). Of the 3 kinds of fluorescent protein investigated, no plasmid was toxic to either cell line. However, sensitivity to lipofection reagents appears to be different; therefore, C2C12 cells may be preferable to MH7A cells when using this transfection system.

## Methods

### Cell culture

Human MH7A synovial cells isolated from the intra-articular soft tissue of the knee joint of RA patients were obtained from Riken (Saitama, Japan). MH7A is a cell line established by transfection with the SV40 T antigen (Miyazawa et al. 1998). MH7A cells were cultured in RPMI 1640 (Sigma, St. Louis, MO) containing 10% heat-inactivated fetal bovine serum (FBS; Whittaker, Walkersville, MD), 100 units/ml of penicillin, and 100 μg/ml of streptomycin (Invitrogen, San Diego, CA).

The C2C12 mouse myoblast cell line was purchased from American Type Culture Collection (Manassas, VA, USA) (13). C2C12 cells were grown in Dulbecco’s Modified Eagle’s Medium (DMEM) (Sigma, St. Louis, MO, USA) containing 10% fetal calf serum (FCS) and antibiotics (100 units/ml penicillin, and 100 μg/ml streptomycin).

Both cell types were cultured in 24-well plates in a CO_2_ incubator EX9100 (Wakenbtech, Kyoto, Japan) in an atmosphere of 5% CO_2_ at 37°C.

### Genetic manipulation

Genes encoding each of 3 different fluorescent proteins of different colors, viz., AcGFP, DsRed, and Strawberry were individually introduced into the multi-cloning site of pcDNA3.1(+) (Invitrogen). The AcGFP-encoding DNA fragment was excised from the plasmid pAcGFP (Clontech, CA, USA) using restriction enzymes *Bam*HI and *Eco*RI. The DsRed- and the Strawberry-encoding fragments were similarly obtained from the plasmids pDsRed-monomer and pmStrawberry (Clontech), respectively. These fragments were inserted into an expression vector for mammalian hosts, the pcDNA3.1(+). The resulting plasmids were named pcDNA-AGF (AcGFP), pcDNA-DRD (DsRed), and pcDNA-STB (mStrawberry). The nucleotide sequences of these constructs were confirmed by sequencing using an ABI PRISM 3100 Genetic Analyzer (Applied Biosystems, Foster City, CA, USA).

Constructs were introduced into mammalian cells for transient expression using a liposome reagent (Felgner and Ringold 1989), following the manufacturer’s instructions for the use of lipofectamine 2000 (Invitrogen). Cells used for transfection were seeded at 1.7 × 10^5^ cells/well in a 24-well plate and cultured for 1 day before transfection.

### Microscopy

Microscopic observation was performed using a fluorescence microscope, BIOREVO BZ-9000 (Keyence, Osaka, Japan), with a band path filter set, viz., BZ filter GFP-BP (470/40, 535/50 nm) for green fluorescence, or BZ filter TRITC (540/25, 605/55 nm) for red fluorescence observations.

### Fluorescent analyses

Before analyses of the fluorescent intensity of cultured cells, the medium in each well was substituted with 500 ml of phosphate buffered saline (PBS). Fluorescence units were measured using the SpectraMax M2 Microplate Reader (Molecular Device, CA, USA), with excitation/emission wave length set at 475 nm/505 nm, 556 nm/586 nm, and 574 nm/596 nm for AcGFP, DsRed, and mStrawberry, respectively. Each measurement was performed at room temperature, and fluorescence intensity was recorded with SoftMax Pro5.2 software. After measurement, the PBS was substituted with medium for further cultivation of the cells.

### Determination of cell viability

Cell viability was determined using a Cell Counting Kit-8 (Dojindo, Kumamoto, Japan), in which 2-(2-methoxy-4-nitrophenyl)-3-(4-nitrophenyl)-5-(2,4-disulfophenyl)-2H-tetrazolium monosodium salt (WST-8) was used as a substrate (Ohuchida et al. 2004). Absorbance was measured using a SpectraMax M2 Microplate Reader (Molecular Device) at 450 nm.
